# Editorial: Women in radiation oncology: 2021

**DOI:** 10.3389/fonc.2023.1162683

**Published:** 2023-03-17

**Authors:** Constanza Martinez, Christina Tsien

**Affiliations:** Department of Radiation Oncology, McGill University Health Centre, Montreal, QC, Canada

**Keywords:** breast cancer, artificial intelligence (AI), stereotactic ablation body radiation therapy, palliation therapy, partial breast irradiation (PBI)

In this special edition of Frontiers in Oncology, we would like to start by thanking all the contributors and congratulating them for their fine efforts. This edition highlights the importance of research mentorship, especially for women in the subspecialty of radiation oncology.

During the recent pandemic, there has been an increased emphasis on innovative ways to improve access to cancer care for women. In this edition, we will highlight papers that aim to shed light on novel advances, including the use of accelerated radiation, stereotactic body radiation therapy (SBRT), and artificial intelligence.

Highly aggressive triple-negative breast cancer (TNBC) is typically treated with neoadjuvant chemotherapy and immunotherapy (1–3). The specific radiobiological characteristics of TNBC tumor cells and their response to different RT fractionation regimens had not been fully elucidated. To address this, Grosche et al. studied the effects of normo-fractionated RT (NormRT) of 50Gy in 2Gy per fraction compared to hypo-fractionated RT (HypoRT) of 40Gy in 2.67Gy per fraction in a normal epithelial breast cancer cell line (MCF10A) and compared these results to those of 2 TNBC BRCA mutant cell lines (HCC1395 and HCC1937). Altogether, these preclinical data support previous studies on the equal effectiveness at the cellular level of NormoRT and HypoRT as tested by this group *in vitro*. The additional use of preclinical models, including patient-derived xenografts, to assess the impact of metabolic and spatial heterogeneity (4) in short- and long-term responses to treatment should also be examined.

The use of accelerated partial breast irradiation (APBI) for younger patients with high-risk features, including the TNBC subtype, continues to be an area of controversy. Goulding et al. reported on a retrospective analysis of 269 patients with high-risk characteristics, including TNBC, ER, tumor size < 3 cm, and age 40-50, who were receiving 38.5 Gy BID in 10 fx. High-risk features, including TNBC and ER histology, were significantly correlated with an increased risk of axillary recurrence. These data highlight the need for further investigation into the use of APBI for younger patients with high-risk features (5).

The benefit of SBRT for breast cancer patients with oligometastatic disease remains an area of continued investigation (6, 7). Lemoine et al. performed a retrospective analysis of the use of SBRT in oligometastatic breast cancer treated with SBRT. In this study, 44 patients were included who had between two and five lesions. The patients had metastatic disease in the bone (44.4%), liver (40.7%), and lung (11.1%). Overall, the results showed high local control, low toxicities, and, in combination with systemic treatment, a progression-free survival (PFS) of greater than 80%.

Vazquez et al. conducted a retrospective analysis of 708 patients who received palliative radiation therapy (RT) for metastatic disease from primary lung (31%), breast (14.8%), and gastrointestinal (14.8%) cancer. Predominantly, palliative RT was delivered to bone metastases (56%), and a single-fraction treatment was used on 34.4% (243) of the patients. The results identified that the 30-day mortality (30-DM) rate was 14.5% (124/708 patients). Importantly, the predictive factor for the 30-DM rate was performance status (ECOG) 2-3 (p= 0.0001). Increased use of single-fraction RT should be taken into consideration when offering palliative radiotherapy compared to the best supportive care. The main objective of treatment—symptom relief—should be discussed with and emphasized to our patients.

Artificial intelligence (AI) is being incorporated into radiation oncology to predict outcomes, improve patient selection, optimize treatment planning, and generate auto-contouring or auto-segmentation tools. Artificial intelligence or machine learning may ultimately be a useful tool for physicians to improve patient selection and treatment. Volpe et al. performed a systematic review of electronic databases that used machine learning (ML) or radiomics specifically in head and neck radiotherapy. They identified 48 studies, including 21 on auto-segmentation, 12 on oncologic outcome prediction, 10 on toxicity prediction, and 4 on treatment planning. Quantitative image features were used in 9/48 studies (19%), and computed tomography was the most used imaging modality in 40% of cases. The clinical applications of AI in radiation oncology continue to increase. The use of CT/MR imaging, auto-segmentation and auto-contouring tools, and virtual reality tools are essential for improving outcomes in radiation oncology.

We hope that our readers enjoy this special edition with its emphasis on women scientists in the field of radiation oncology and that they are inspired to work together and support the mentorship of young investigators in this field. Embracing novel advances and tools is essential for improving outcomes in radiation oncology. Training our future oncologists is a necessary factor in the continued value and importance of radiation oncology in improving cancer care outcomes for women. We have a strong group of talented investigators, and the future of our field remains bright.

## Author contributions

Writing and editing the editorial CM, CT, review editorial. All authors contributed to the article and approved the submitted version.
